# Immune cells markers within local tumor microenvironment are associated with EBV oncoprotein in nasopharyngeal cancer

**DOI:** 10.1186/s12885-022-09948-9

**Published:** 2022-08-13

**Authors:** Soehartati A. Gondhowiardjo, Marlinda Adham, Lisnawati Rachmadi, Tubagus Djumhana Atmakusuma, Demak Lumban Tobing, Mahesa Auzan, Agustinus Darmadi Hariyanto, Dede Sulaeman, Tiara Bunga Mayang Permata, Handoko

**Affiliations:** 1 Department of Radiation Oncology, Faculty of Medicine, Universitas Indonesia / Cipto Mangunkusumo National General Hospital, Jakarta – Indonesia, Jl. Salemba Raya No. 6, Jakarta, Indonesia 10430; 2 Department of ENT, Faculty of Medicine, Universitas Indonesia / Cipto Mangunkusumo National General Hospital, Jakarta – Indonesia, Jl. Salemba Raya No. 6, Jakarta, Indonesia 10430; 3 Department of Anatomical Pathology, Faculty of Medicine, Universitas Indonesia / Cipto Mangunkusumo National General Hospital, Jakarta – Indonesia, Jl. Salemba Raya No. 6, Jakarta, Indonesia 10430; 4 Department of Medical Oncology, Faculty of Medicine, Universitas Indonesia / Cipto Mangunkusumo National General Hospital, Jakarta – Indonesia, Jl. Salemba Raya No. 6, Jakarta, Indonesia 10430; 5 Department of Clinical Pathology, Dharmais National Cancer Hospital, Jakarta – Indonesia, Jl. Letjen S. Parman No. 84-86, Jakarta, Indonesia 11420

**Keywords:** Nasopharyngeal cancer, EBV oncoproteins, Immune markers, Tumor progression, Microenvironment

## Abstract

**Introduction:**

EBV infection in nasopharyngeal cancer ensued in latent infection mode. In this latent infection various EBV oncoproteins such as EBNA1 and LMP1 was expressed. EBV oncoproteins could theoretically recruit immune cells, which might help to control cancer. Therefore, this study was aimed to elucidate the association with EBV oncoproteins (EBNA1 and LMP1), immune markers (CD4, CD8, and FOXP3) from nasopharyngeal cancer microenvironment with tumor progression.

**Method:**

Nasopharyngeal biopsy was obtained from patients suspected to have nasopharyngeal cancer. Those samples with microscopically confirmed nasopharyngeal cancer were tested for EBNA1, LMP1, CD4, CD8, and FOXP3 concentration with ELISA, then verified with IHC. Each patient tumor volume was assessed for primary nasopharyngeal tumor volume (GTVp) and neck nodal metastases tumor volume (GTVn). Correlation test with Spearman correlation and scatterplot were carried out.

**Result:**

Total 23 samples with nasopharyngeal cancer were analyzed. There was moderate correlation (*ρ* = 0.45; *p* value = 0.032) between LMP1 and GTVp. There was strong correlation (*ρ* = 0.81; *p* value < 0.001) between CD8 and GTVp. There was also moderate correlation (*ρ* = 0.6; *p* value = 0.002) between FOXP3 and GTVp. The CD8 concentration has moderate correlation with both EBNA1 (*ρ* = 0.46; *p* value = 0.026) and LMP1 (*ρ* = 0.47; *p* value = 0.023). While FOXP3 has moderate correlation with only LMP1 (*ρ* = 0.58; *p* value = 0.004). No correlation was found between all the markers tested here with GTVn.

**Discussion:**

We found larger primary nasopharyngeal tumor was associated with higher CD8 marker. This was thought due to the presence of abundance CD8 T cells in the nasopharynx, but those abundance CD8 T cells were suspected to be dysfunctional. The nasopharyngeal cancer was also known to upregulate chemokines that could recruit T regulatory FOXP3 cells. Furthermore, T regulatory FOXP3 cells differentiation was induced through several pathways which was triggered by EBNA1. The correlation found in this study could guide further study to understand nasopharyngeal carcinogenesis and the relationship with our immune system.

## Key points

The immune markers CD8 and FOXP3 correlated with EBNA1 or LMP1 in clinical nasopharyngeal specimens. Furthermore, all those markers were associated with nasopharyngeal cancer progression at least locally.

## Introduction

Nasopharyngeal cancer, especially the non-keratinized nasopharyngeal cancer, is one of the cancer that is related to viral Epstein Barr Virus (EBV) infection [1, 2]. In Southern Asia and South East Asia, where nasopharyngeal cancer is endemic [1, 3]. EBV is almost always detected in nasopharyngeal cancer specimen [4, 5]. Due to the association of EBV with nasopharyngeal cancer, EBV viral load has been studied as a potential biomarker for screening, diagnosis, until predicting prognosis [4, 6–9]. There was some evidence suggesting that higher EBV viral load was associated with worse prognosis such as greater risk of relapse [8, 9]. However, the initial or pre-treatment EBV viral load was not shown to be correlated with tumor progression or tumor stage [8]. A recent study by our group showed that though EBV was presence, but the presence of EBV within the nasopharyngeal cancer sample has no association with the cancer progression [4].

The discordant finding between EBV viral load with tumor progression has led us to reinvigorate the possibility of EBV products as the driver of nasopharyngeal cancer progression. Various latent EBV products have been known to implicate in EBV associated carcinogenesis [10–12]. We thought that EBV products that drive the cancer progression should have better association with tumor progression compared to EBV viral load only. A study examining plasma level of various EBV products have reaffirmed the association between those EBV products with risk of metastasis [13]. We, therefore, speculated that the detection of those EBV proteins directly from nasopharyngeal cancer sample could confer a stronger predictive value.

Furthermore, we have shown that the density of tumor infiltrate within nasopharyngeal cancer tumor microenvironment was also associated with tumor progression [5]. Various data has indicated that the immune contexture within tumor microenvironment in most solid cancer has a great role predicting treatment outcomes [14–18]. For instance, higher density of infiltrating tumor T regulatory FOXP3 cells was shown to negatively affect prognosis in breast cancer [19]. Lower expression of FOXP3 was found to confer better clinical outcome in melanoma sample [20]. While higher CD8 T cell infiltration in breast cancer was found to be associated with significant reduction of death from breast cancer [21]. Another study also found higher CD8 infiltration post treatment to be associated with favorable prognosis in non-small cell lung cancer [22]. In oral squamous cell carcinoma, denser CD8 tumor infiltration also found to confer better prognosis [23].

Therefore, we aimed to elucidate the association between EBV oncoproteins and various immune markers within tumor microenvironment with tumor progression in nasopharyngeal cancer. We selected two EBV oncoproteins that has been known to implicate in nasopharyngeal carcinogenesis, which are Epstein-Barr nuclear antigen 1 (EBNA1) and latent membrane protein 1 (LMP1). We also measured the immune markers CD4 (T helper cell), CD8 (cytotoxic T cell), and FOXP3 (T regulatory cell) within the nasopharyngeal cancer tumor microenvironment.

## Methods

### Study design and patient recruitment

This study was a prospective study that recruited patient from a single academic hospital in Indonesia. This study has passed ethical clearance from the Faculty of Medicine, Universitas Indonesia ethical committee. All patients that were suspected to have nasopharyngeal cancer were approached for inform consent between July 2019 to June 2021. All patients have the full right to choose whether to participate or not in this study. The researchers have ensured those who declined to participate will receive treatment similarly with those who participated according to the hospital protocol. Those who participated in this study did not receive any monetary compensation.

The patients participated in this study have their nasopharyngeal biopsy procedure as usual as per hospital protocol, with an extra biopsy taken for the purpose of this study. All biopsied specimens for this study was initially stored in -80 °C in liquid nitrogen tank before the laboratory examination was done. Those with positive histopathological diagnosis of nasopharyngeal cancer were retrieved from the storage and underwent laboratory examination.

### Sample preparation

All 23 samples were retrieved from the frozen nitrogen tank storage, then thawed in room temperature for 30 min. The thawed sample were cut until it weighted around 50 mg. This small 50 mg sample was the continued to be processed. Then it was further sliced with a sterile scalpel until the whole sample disintegrated. A Phosphate-buffered saline (PBS) was added to the disintegrated sample until 500 µl. The sample was then homogenized with an ultrasonic disruptor until there was no solid specimen presence. This homogenized sample was ready for further testing with respective antibodies.

### EBV Oncoproteins and immune markers examination

All EBV oncoproteins and immune markers used in this study underwent Enzyme-Linked Immunosorbent Assay (ELISA) test. The EBNA1 antibody used was EBNA1 IgG ELISA kit from DRG. The concentration tested from this EBNA1 ELISA kit was a relative concentration of the tested sample relative to the controlled sample from the kit, which was expressed as DU unit (as per manufacturer guideline). The LMP1 antibody used was LMP1 ELISA MBS721506 kit from MyBiosource. The CD4 antibody, CD8 antibody, and FOXP3 antibody used were CD4 ELISA MBS2702976 kit, CD8 ELISA MBS165145 kit, FOXP3 ELISA MBS162054 kit, respectively, all from MyBiosource. The LMP1, CD4, CD8, and FOXP3 ELISA kit all measured absolute concentration, which was expressed in ng unit.

The homogenized sample underwent ELISA test for each antibody with their respective kits according the manufacturer guidelines. The protein content was then detected using spectrophotometer with the recommended wave length based on each kit’s guideline. All samples were also tested for total protein content using Bradford protein assay. The final protein concentration of each marker was calculated by dividing the protein content of each marker by the total protein content based on Bradford protein assay. This method enabled an objective comparison of each marker between samples.

### Immunohistochemistry (IHC) confirmation of immune markers

All immune markers tested in this study, CD4, CD8, FOXP3, were confirmed through IHC. All paraffin blocks of those patients with confirmed nasopharyngeal cancer were retrieved. Three additional cuts with thickness of 3 µm was done from the paraffin blocks. They were placed on glass slides. Then, they were deparaffinized with Xylol and rehydrated with alcohol. Furthermore, the slide was treated with Tris–EDTA on decloaking chamber. They were rinsed with Phosphate Buffer Saline (PBS) and stained with primary IHC antibody of CD4 (Biocare), CD8 (Biocare), and FOXP3 (GeneTex) for each slide, respectively. Then they were re-rinsed with PBS and counterstained with hematoxylin stain. Then the slides were rehydrated again in alcohol and cleared with Xylol.

The IHC slides were evaluated for the presence of the immune markers on the immune cells. Staining on the immune cells’ membrane should be presence for CD4 and CD8. While staining within nuclei of the immune cells should be presence for FOXP3. This IHC evaluation was to confirm the detected CD4, CD8, and FOXP3 from ELISA, was indeed derived from the immune cells. Evaluation was done using Olympus BX50 microscope with 200 X magnification. Representative section of IHC images were captured and then transferred to QuPath software version 0.2.3 for better cell to cell evaluation. This IHC examination was done and then evaluated by a senior pathologist.

### Tumor staging and tumor volume determination

All patients were staged with nasopharyngeal CT scan or nasopharyngeal MRI with contrast agent, chest X-Ray, abdominal ultrasound, and bone scan. The nasopharyngeal CT Scan or MRI images was always obtained from frontal sinus until lower neck. The staging used was based on American Joint Committee on Cancer (AJCC) version 8. The nasopharyngeal CT scan or nasopharyngeal MRI which was conducted within 1-month time from the date of nasopharyngeal biopsy was used as the basis of tumor volume determination.

The tumor volume was measured separately between primary tumor and neck nodes. The tumor volume based on nasopharyngeal CT scan or MRI was delineated by an experienced radiation oncologist with Eclipse software from Varian. The primary nasopharyngeal tumor volume was denoted GTVp, while the nodal tumor volume was denoted GTVn.

### Statistical tests

All the markers (EBV oncoproteins and immune markers) and the tumor volume were numeric variable. A correlation test was done for all variables using Spearman correlation test. For graphical presentation, a scatterplot was used to display the correlation data of all tested variables. All data was processed with IBM SPSS Statistic Software version 25.

## Results

### Patient characteristics

A total 23 samples were tested and analyzed. All 23 samples have histopathological diagnosis of non-keratinized undifferentiated nasopharyngeal carcinoma. Mean age of the whole patient cohort was 45.6 years old with range of 31–63 years old. Majority of patient was male 60.9%. The distribution of patient TNM staging was available on Table 1. There was no early stage I localized nasopharyngeal cancer in this patient cohort. The median volume of primary nasopharynx tumor (GTVp) and nodal tumor volume (GTVn) were 41.4 mm^3^ and 40.1 mm^3^ respectively.

**Table 1:** Patient characteristics (*n* = 23 samples)

Age (mean ± SD)	45.65 ± 7.9 years old
Gender	
Male	14 (60.9%)
Female	9 (39.1%)
T Stage	
T1	2 (8.7%)
T2	11 (47.8%)
T3	3 (13%)
T4	7 (30.4%)
N Stage	
N0	1 (4.3%)
N1	2 (8.7%)
N2	7 (30.4%)
N3	13 (56.5%)
M Stage	
M0	22 (95.7%)
M1	1 (4.3%)
Anatomical Stage	
Stage II	1 (4.3%)
Stage III	6 (26.1%)
Stage IVA	15 (65.2%)
Stage IVB	1 (4.3%)
GTVp (median, range)	41.4 (13.2—128.8) mm^3^
GTVn (median, range)	40.1 (1.2—633.5) mm^3^

### EBV Oncoproteins association with tumor progression

**Fig. 1:**
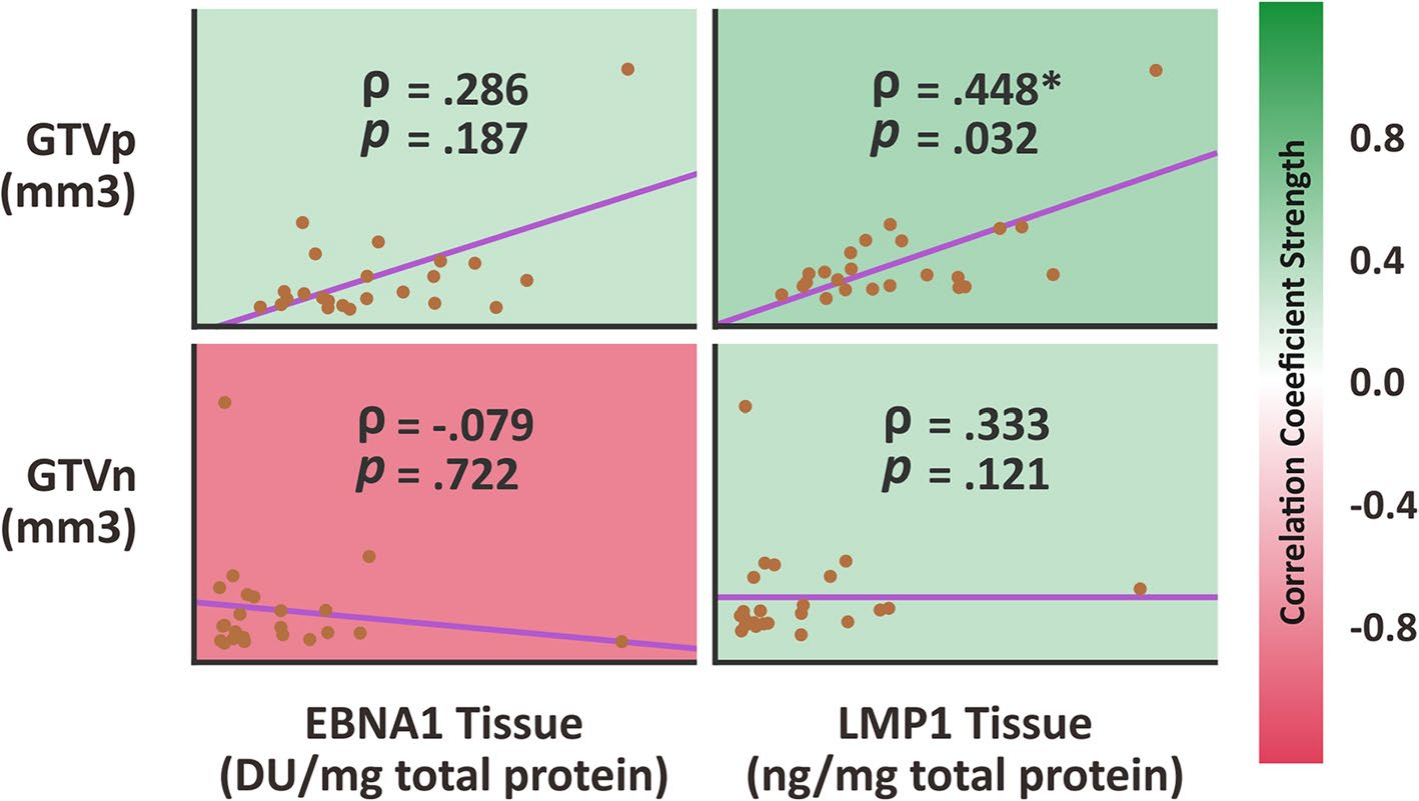
Scatterplot showing correlation between both EBNA1 and LMP1 from local nasopharyngeal biopsy versus GTVp and GTVn volume. The strength of correlation was depicted with color

The median value of EBNA1 and LMP1 oncoproteins concentration detected from local primary nasopharyngeal cancer specimen were 46.11 DU/mg total protein and 0.75 ng/mg total protein, respectively (Table 2). There was a moderate correlation between LMP1 concentration from local primary nasopharyngeal cancer specimen with GTVp with ρ Spearman correlation coefficient of 0.45 (*p* value = 0.032). The increased in size of primary nasopharyngeal tumor (GTVp) was associated with the increased of LMP1 concentration from local primary nasopharyngeal cancer specimen, Fig. 1. However, EBNA1 concentration from local primary nasopharyngeal cancer specimen did not correlate with the GTVp (ρ Spearman correlation coefficient of 0.29; *p* value = 0.187).

**Table 2:** EBV Oncoproteins and immune markers concentration from local primary nasopharyngeal cancer specimen (*n* = 23 samples)

EBNA1 Protein (median, range)	46.11 (7.48—600.02) DU / mg total protein
LMP1 Protein (median, range)	0.75 (0.26—5.08) ng / mg total protein
CD4 Protein (median, range)	0.099 (0.04—1.31) ng / mg total protein
CD8 Protein (median, range)	4983.47 (3150.11—35,842.03) ng / mg total protein
FOXP3 Protein (median, range)	66.45 (16.67—580.34) ng / mg total protein

The LMP1 concentration from local primary nasopharyngeal cancer specimen did not correlate with GTVn (ρ Spearman correlation coefficient of 0.33; *p* value = 0.121). Similarly, the EBNA1 concentration from local primary nasopharyngeal cancer specimen also did not correlate with GTVn (ρ Spearman correlation coefficient of -0.08; *p* value = 0.722), Fig. 1. Both LMP1 and EBNA1 detected from local primary nasopharyngeal cancer specimen seemed did not influence the progressivity of nodal metastasis in nasopharyngeal cancer.

### Immune markers association with tumor progression

**Fig. 2:**
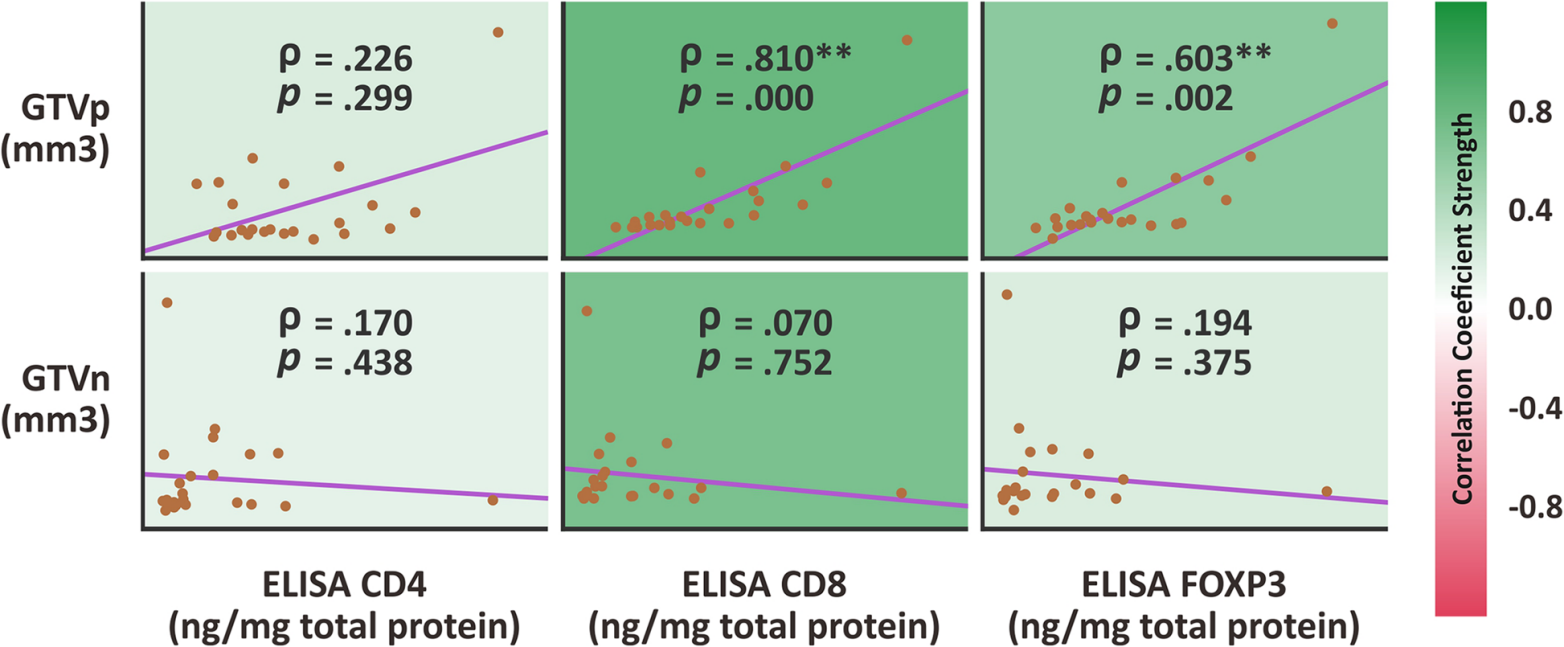
Scatterplot showing correlation between CD4, CD8, and FOXP3 concentration marker from local nasopharyngeal biopsy versus GTVp and GTVn volume. The strength of correlation was depicted with color

The median value of CD4, CD8, and FOXP3 concentration from local primary nasopharyngeal cancer specimen were 0.099, 4983.47, and 66.45 ng / mg total protein, respectively, Table 2. The CD4 concentration from local primary nasopharyngeal cancer specimen did not relate with GTVp volume (ρ Spearman correlation coefficient of 0.23; *p* value = 0.299) nor GTVn volume (ρ Spearman correlation coefficient of 0.17; *p* value = 0.438), Fig. 2.

The CD8 concentration from local primary nasopharyngeal cancer specimen was strongly related to GTVp volume (ρ Spearman correlation coefficient of 0.81; *p* value < 0.001). Larger primary nasopharyngeal tumor was strongly associated with higher concentration of CD8 markers in local primary nasopharynx. While the CD8 concentration from local primary nasopharyngeal cancer specimen had no correlation with GTVn (ρ Spearman correlation coefficient of 0.07; *p* value = 0.752), Fig. 2.

The FOXP3 concentration from local primary nasopharyngeal cancer specimen was moderately associated with GTVp volume (ρ Spearman correlation coefficient of 0.6; *p* value = 0.002). Larger primary nasopharynx tumor was found to be related to higher concentration of FOXP3 markers in local primary nasopharynx. The FOXP3 concentration from local primary nasopharyngeal cancer specimen had no correlation with GTVn (ρ Spearman correlation coefficient of 0.19; *p* value = 0.375), Fig. 2.

**Fig. 3:**
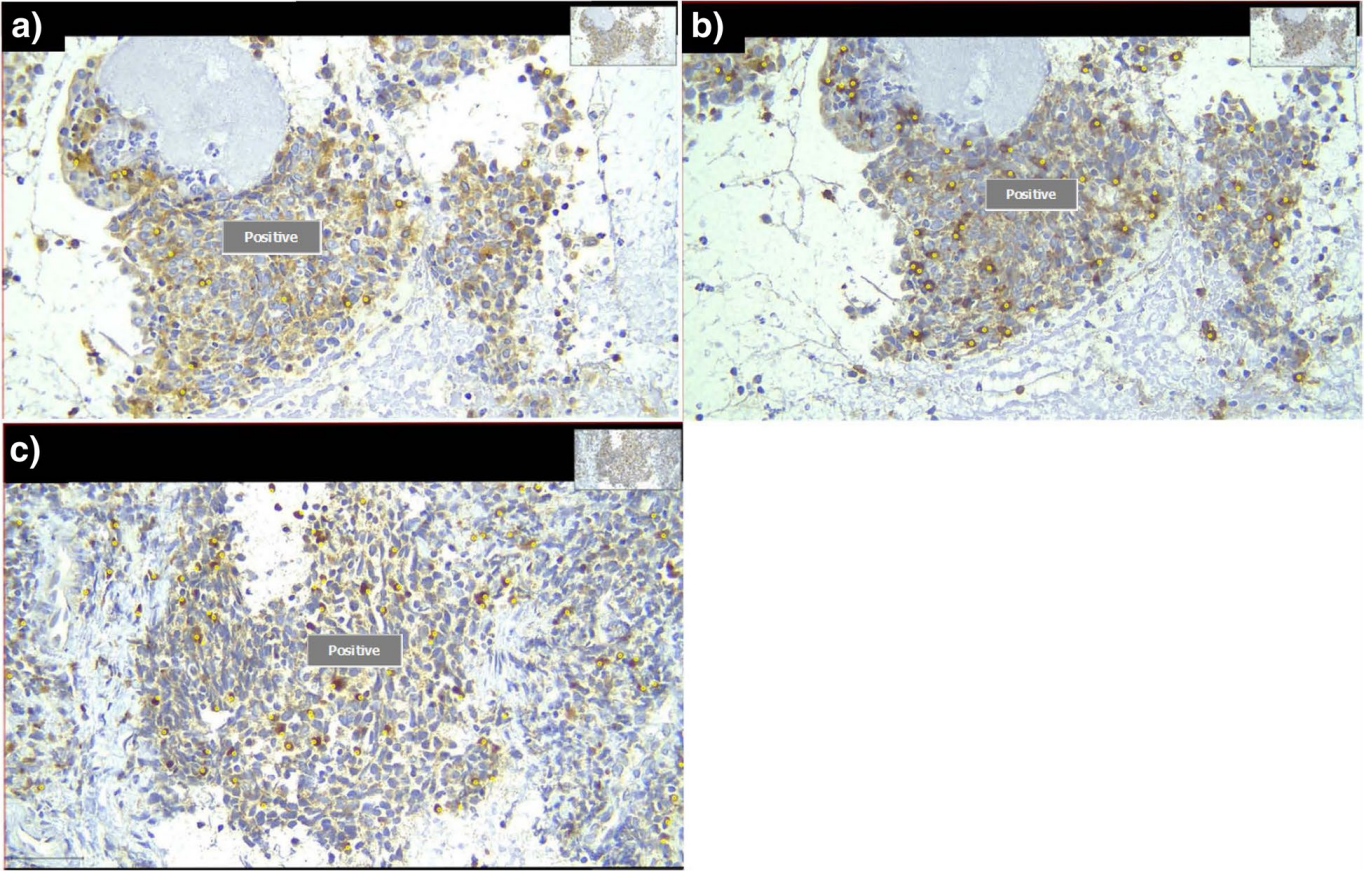
IHC from local nasopharyngeal biopsy showing staining of **a**) CD8 on cell membrane **b**) CD4 on cell membrane **c**) FOXP3 in nucleus (yellow circle indicating the designated immune cells with their respective markers). Image was captured from QuPath software in standard resolution

The CD4, CD8, and FOXP3 markers presence on immune cells were confirmed through IHC examination. The representative IHC slides for each immune markers were shown here, Fig. 3. The immune cells within tumor microenvironment from all samples were confirmed to express CD4 and CD8 on immune cell membranes, and FOXP3 on nucleus of immune cells by IHC examination. This IHC examination, thus validating the immune markers presence on viable immune cells within local tumor microenvironment.

### Immune markers association with EBV oncoproteins

**Fig. 4:**
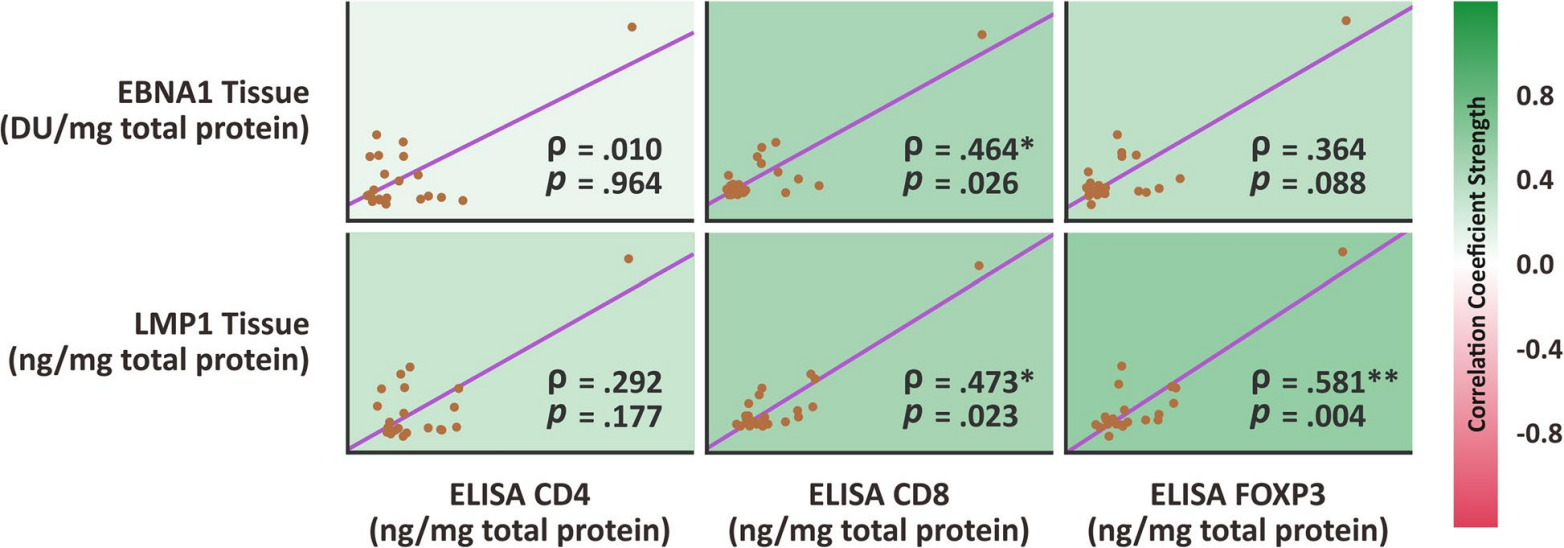
Scatterplot showing correlation between CD4, CD8, and FOXP3 concentration marker versus EBNA1 and LMP1 from local nasopharyngeal biopsy. The strength of correlation was depicted with color

The CD4 concentration from local primary nasopharyngeal cancer specimen has no association with both EBNA1 oncoprotein (ρ Spearman correlation coefficient of 0.01; *p* value = 0.964) and LMP1 oncoprotein (ρ Spearman correlation coefficient of 0.29; *p* value = 0.177) from local primary nasopharyngeal cancer specimen, Fig. 4.

The CD8 concentration from local primary nasopharyngeal cancer specimen has moderate correlation with both EBNA1 oncoprotein (ρ Spearman correlation coefficient of 0.46; *p* value = 0.026) and LMP1 oncoprotein (ρ Spearman correlation coefficient of 0.47; *p* value = 0.023) from local primary nasopharyngeal cancer specimen, Fig. 4. The higher concentration of both EBNA1 and LMP1 from local primary nasopharyngeal cancer specimen were associated with increased concentration of CD8 marker detected from local primary nasopharyngeal cancer specimen.

The FOXP3 concentration from local primary nasopharyngeal cancer specimen has no association with EBNA1 oncoprotein (ρ Spearman correlation coefficient of 0.36; *p* value = 0.088). However, the FOXP3 concentration from local primary nasopharyngeal cancer specimen has moderate correlation with LMP1 oncoprotein concentration (ρ Spearman correlation coefficient of 0.58; *p* value = 0.004) from local primary nasopharyngeal cancer specimen, Fig. 4. Higher LMP1 concentration from local primary nasopharyngeal cancer specimen was related with higher FOXP3 concentration from local primary nasopharyngeal cancer specimen.

## Discussion

The EBNA1 protein is an important protein involved in EBV associated carcinogenesis because this latent EBV protein EBNA1 is always expressed throughout all forms of EBV latency infection. This EBNA1 protein has been known for its role of maintaining the EBV genomes in tumor cells [24, 25]. However, its role directly affecting immortalization or enhancing proliferation of cancer cells are unclear [24]. Nevertheless, this EBNA1 is a prerequisite for continuous integrity of EBV genomes inside host cells, thus making it a potential biomarker in studying cancer progression.

Though EBNA1 has an essential role in EBV associated malignancy, but our study has showed that it did not influence tumor progression in nasopharyngeal cancer. The increased EBNA1 concentration found in local primary nasopharyngeal specimen did not correlate with the progression of primary nasopharyngeal cancer nor the progression of its nodal metastasis. Perhaps, this finding was due to the indirect role of EBNA1 in promoting carcinogenesis [26]. For instance, EBNA1 promoted metastasis, but indirectly through upregulation of various epithelial-mesenchymal transition (EMT) mediators [26]. Therefore, in clinical specimen, EBNA1 was not a powerful predictor of tumor progression.

The LMP1 has been known as the primary EBV oncogene that drive carcinogenesis for decades [11, 27]. LMP1 drives proliferation, causes immortalization, affects immune modulation, promotes angiogenesis, increases genomic instability and modulates cell migration [27, 28]. Our finding further confirmed the established role of LMP1 in promoting carcinogenesis in clinical nasopharyngeal carcinoma sample.

The CD4 marker from local primary nasopharyngeal cancer specimen was not associated with both tumor progressions in primary and nodal site. This finding was not surprising because CD4 was a generic marker expressed in an array of immune cells, from T helper cells, monocytes, macrophages, until dendritic cells. Therefore, the presence of CD4 marker within local tumor microenvironment might implicate a wide range of scenarios. It was not necessary correlate with the process of cancer immune escape nor cancer immune surveillance.

The CD8 marker from local primary nasopharyngeal cancer specimen, in another hand, was found to be positively and significantly related to tumor progressions in primary site. The CD8 cytotoxic T cells are implicated in cellular defense and have important role in cancer immunotherapy [14]. Various evidence in numerous solid human cancer has proved a beneficial role of CD8 cells within tumor microenvironment [21–23, 29]. However, we argued that the larger primary nasopharyngeal tumor that was associated with higher CD8 marker was perhaps due to abundant but dysfunctional CD8 T cells within tumor microenvironment. Though our study was not designed prove it, but there was evidence showing that local nasopharyngeal cancer CD8 T cells failed to produce interferon- γ thus blunting their cytotoxic activity [30]. Another evidence also proved the significant decreased of EBNA1 specific CD8 T cells in patients with nasopharyngeal cancer [31].

Nevertheless, there was perhaps another more trivial explanation regarding the association of higher CD8 marker with larger primary nasopharyngeal tumor. The nasopharyngeal mucosa is part of nasal or nasopharynx associated lymphoid tissue (NALT). Animal study has found that nasal mucosa and NALT has high resident CD8 T cells [32]. Apart of that, we found that higher LMP1 and EBNA1 concertation was related to higher CD8 marker from local primary nasopharyngeal cancer specimen. Both of these EBV oncoproteins are commonly expressed during EBV latent infection. This latent infection mode is the main mode of EBV infection in nasopharyngeal cancer [33]. The latent EBV infection was known to result in memory CD8 T cell retention locally in lymphoid organs, [34, 35] thus further supporting the higher CD8 marker found locally in nasopharyngeal cancer specimen.

The FOXP3 marker from local primary nasopharyngeal cancer specimen was also positively related to tumor progressions in primary site. This FOXP3 marker was known to be a marker of T regulatory cells, an important T cells subset that work to suppress inflammation and has been implicated to promote cancer immune escape in multiple cancer [14, 36]. There was evidence showing that EBNA1 might be associated with the induction of naïve T cells to differentiate into T regulatory cells in nasopharyngeal cancer [37]. This induction of T regulatory cells differentiation was proven due to the expression of TGF‐β1, which was shown to have positive correlation with EBNA1 expression [37].

Apart of that, the chemokine CCL20 was overexpressed in nasopharyngeal cancer [37]. Blockage of this CCL20 with monoclonal antibody has been shown to abrogate T regulatory migration [37]. Thus, it can be inferred that naturally nasopharyngeal cancer would express higher CCL20 chemokine which stimulated migration of T regulatory cells, further amplifying the progression of nasopharyngeal cancer. Thus, all of these findings further supported the possibility of higher FOXP3 markers in larger primary nasopharyngeal cancer.

The LMP1 was also found to have positive association with FOXP3 concentration from local primary nasopharyngeal cancer specimen. The LMP1 has been shown experimentally to induce IL-10 production [38]. High IL-10 concentration together with presence of T regulatory cells suppressed T cell responses, [38] thereby promoting nasopharyngeal cancer progression [39]. The positive LMP1 correlation found with local FOXP3 marker concentration from primary nasopharyngeal cancer specimen was perhaps due to indirect effect of both LMP1 and FOXP3 in promoting tumor progression, instead of the direct effect of LMP1 toward T regulatory cells.

In our study, the EBNA1 had no correlation with FOXP3 concentration from local primary nasopharyngeal cancer specimen. This was perhaps due to a more indirect role of EBNA1 in promoting tumor progression and stimulating T regulatory cells. There was evidence indicating the role of EBNA1 in attracting T regulatory cells, but it was a lengthy pathway involving upregulation of the (TGFβ1)-SMAD3-PI3K-AKT-c-JUN-CXCL12-CXCR4 axis and downregulation of miR-200a [40, 41]. Therefore, EBNA1 per se was not seen to correlate with FOXP3 from clinical nasopharyngeal sample.

The negative correlation found between all the local immune markers and EBV oncoproteins concentration with nasopharyngeal cancer nodal tumor volume was as expected. Immune infiltrates of tumor in primary and metastatic site has been shown to be completely different [42]. Though cancer may originate from an abnormal clone, but along the way, the tumor might exhibit different genotype and phenotype different from their initial ancestors. This explained various failure in cancer treatment. Nevertheless, understanding tumor microenvironment is always necessary to understand the biology of tumor evolution and its interaction with host immune response, thereby allowing us to tailor a better strategy to treat cancer in the future.

## Data Availability

Not applicable.

## Abbreviations

EBV: Epstein-Barr Virus
EBNA1: Epstein–Barr Nuclear Antigen 1
LMP1: Latent Membrane Protein 1
FOXP3: Fork Head Box P3
CD4: Cluster of Differentiation 4
CD8: Cluster of Differentiation 8
ELISA: Enzyme-Linked Immunosorbent Assay
IHC: Immunohistochemistry
GTVp: Gross Tumor Volume primary
GTVn: Gross Tumor Volume node
IL-10: Interleukin-10
CCL20: (C–C motif) ligand 20
NALT: Nasopharynx Associated Lymphoid Tissue

## References

[1] Chen Y-P, Chan ATC, Le Q-T, Blanchard P, Sun Y, Ma J (2019). Nasopharyngeal carcinoma. Lancet (London, England).

[2] Gondhowiardjo SA, Adham M, Lisnawati L, et al. Current Immune-Related Molecular Approach in Combating Nasopharyngeal Cancer. World J Oncol Vol 10, No 4–5, Oct 2019. 2019 Sep; 10.14740/wjon1214 PMC6785271 31636788

[3] Gondhowiardjo S, Muthalib A, Khotimah S, Rachman A. Nimotuzumab combined with radiotherapy reduces primary tumor and nodal volume in advanced undifferentiated nasopharyngeal carcinoma. Vol. 5, Asia-Pacific Journal of Clinical Oncology. 2009. p. 175.

[4] Gondhowiardjo SA, Handoko, Adham M, et al. Epstein–Barr Virus (EBV) Viral Load in Tumor Cells Did Not Predict Tumor Extensiveness in Nasopharyngeal Cancer. Microbiol Res (Pavia). 2021;12(1):150–6.

[5] Gondhowiardjo SA, Handoko, Adham M, et al. Tumor microenvironment predicts local tumor extensiveness in PD-L1 positive nasopharyngeal cancer. PLoS One. 2020;15(3):e0230449. 10.1371/journal.pone.0230449 PMC7082005 32191754

[6] Chan KCA, Woo JKS, King A (2017). Analysis of Plasma Epstein-Barr Virus DNA to Screen for Nasopharyngeal Cancer. N Engl J Med.

[7] Adham M, Greijer AE, Verkuijlen SAWM, et al. Epstein-Barr Virus DNA Load in Nasopharyngeal Brushings and Whole Blood in Nasopharyngeal Carcinoma Patients before and after Treatment. Clin Cancer Res. 2013;2175–87. 10.1158/1078-0432.CCR-12-2897 23493345

[8] Twu C-W, Wang W-Y, Tsou H-H (2020). Effects of Epstein-Barr virus viral load and different treatment modality for stage III nasopharyngeal carcinoma. Head Neck.

[9] Nilsson JS, Forslund O, Andersson FC, Lindstedt M, Greiff L (2019). Intralesional EBV-DNA load as marker of prognosis for nasopharyngeal cancer. Sci Rep.

[10] Richardo T, Prattapong P, Ngernsombat C, et al. Epstein-Barr Virus Mediated Signaling in Nasopharyngeal Carcinoma Carcinogenesis. Cancers (Basel). 2020 Aug;12(9). 10.3390/cancers12092441 PMC7565514 32872147

[11] Gondhowiardjo S (2000). Epstein-Barr virus latent membrane protein 1 (EBV-LMP1) and tumor proliferation rate as predictive factors of nasopharyngeal cancer (NPC) radiation response. Gan To Kagaku Ryoho.

[12] Elgui de Oliveira D, Muller-Coan BG, Pagano JS. Viral Carcinogenesis Beyond Malignant Transformation: EBV in the Progression of Human Cancers. Trends Microbiol. 2016 Aug;24(8):649–64. 10.1016/j.tim.2016.03.008 PMC5489061 27068530

[13] Sun L, Wang Y, Shi J, Zhu W, Wang X (2020). Association of Plasma Epstein-Barr Virus LMP1 and EBER1 with Circulating Tumor Cells and the Metastasis of Nasopharyngeal Carcinoma. Pathol Oncol Res.

[14] Gondhowiardjo SA, Handoko, Jayalie VF, et al. Tackling Resistance to Cancer Immunotherapy: What Do We Know? Molecules. 2020 Sep;25(18). 10.3390/molecules25184096 PMC7570938 32911646

[15] Sideras K, Galjart B, Vasaturo A (2018). Prognostic value of intra-tumoral CD8(+) /FoxP3(+) lymphocyte ratio in patients with resected colorectal cancer liver metastasis. J Surg Oncol.

[16] Ono T, Azuma K, Kawahara A (2020). Predictive value of CD8/FOXP3 ratio combined with PD-L1 expression for radiosensitivity in patients with squamous cell carcinoma of the larynx receiving definitive radiation therapy. Head Neck.

[17] Li F, Zhao Y, Wei L, Li S, Liu J (2018). Tumor-infiltrating Treg, MDSC, and IDO expression associated with outcomes of neoadjuvant chemotherapy of breast cancer. Cancer Biol Ther.

[18] Darwis NDM, Oike T, Kubo N, Gondhowiardjo SA, Ohno T. Characteristics of PSA Bounce after Radiotherapy for Prostate Cancer: A Meta-Analysis. Cancers (Basel). 2020 Aug;12(8). 10.3390/cancers12082180 PMC7465291 32764448

[19] Takenaka M, Seki N, Toh U (2013). FOXP3 expression in tumor cells and tumor-infiltrating lymphocytes is associated with breast cancer prognosis. Mol Clin Oncol.

[20] Gerber AL, Münst A, Schlapbach C (2014). High expression of FOXP3 in primary melanoma is associated with tumour progression. Br J Dermatol.

[21] Ali HR, Provenzano E, Dawson S-J (2014). Association between CD8+ T-cell infiltration and breast cancer survival in 12,439 patients. Ann Oncol Off J Eur Soc Med Oncol.

[22] Yoneda K, Kuwata T, Kanayama M (2019). Alteration in tumoural PD-L1 expression and stromal CD8-positive tumour-infiltrating lymphocytes after concurrent chemo-radiotherapy for non-small cell lung cancer. Br J Cancer.

[23] Shimizu S, Hiratsuka H, Koike K (2019). Tumor-infiltrating CD8(+) T-cell density is an independent prognostic marker for oral squamous cell carcinoma. Cancer Med.

[24] Frappier L (2012). Role of EBNA1 in NPC tumourigenesis. Semin Cancer Biol.

[25] Hau PM, Lung HL, Wu M (2020). Targeting Epstein-Barr Virus in Nasopharyngeal Carcinoma. Front Oncol.

[26] Wang L, Tian W-D, Xu X (2014). Epstein-Barr virus nuclear antigen 1 (EBNA1) protein induction of epithelial-mesenchymal transition in nasopharyngeal carcinoma cells. Cancer.

[27] Kieser A, Sterz KR (2015). The Latent Membrane Protein 1 (LMP1). Curr Top Microbiol Immunol.

[28] Wakae K, Kondo S, Pham HT (2020). EBV-LMP1 induces APOBEC3s and mitochondrial DNA hypermutation in nasopharyngeal cancer. Cancer Med.

[29] Tsiatas M, Kalogeras KT, Manousou K (2018). Evaluation of the prognostic value of CD3, CD8, and FOXP3 mRNA expression in early-stage breast cancer patients treated with anthracycline-based adjuvant chemotherapy. Cancer Med.

[30] Li J, Zeng X, Mo H (2007). Functional Inactivation of EBV-Specific T-Lymphocytes in Nasopharyngeal Carcinoma: Implications for Tumor Immunotherapy. PLoS ONE.

[31] Fogg MH, Wirth LJ, Posner M, Wang F (2009). Decreased EBNA-1-specific CD8+ T cells in patients with Epstein-Barr virus-associated nasopharyngeal carcinoma. Proc Natl Acad Sci U S A.

[32] Rodríguez-Monroy MA, Rojas-Hernández S, Moreno-Fierros L (2007). Phenotypic and functional differences between lymphocytes from NALT and nasal passages of mice. Scand J Immunol.

[33] Tsao SW, Tsang CM, Lo KW. Epstein-Barr virus infection and nasopharyngeal carcinoma. Philos Trans R Soc London Ser B, Biol Sci. 2017 Oct;372(1732). 10.1098/rstb.2016.0270 PMC5597737 28893937

[34] Long HM, Meckiff BJ, Taylor GS (2019). The T-cell Response to Epstein-Barr Virus-New Tricks From an Old Dog. Front Immunol.

[35] Woon HG, Braun A, Li J (2016). Compartmentalization of Total and Virus-Specific Tissue-Resident Memory CD8+ T Cells in Human Lymphoid Organs. PLOS Pathog.

[36] Zhang Y-L, Li J, Mo H-Y (2010). Different subsets of tumor infiltrating lymphocytes correlate with NPC progression in different ways. Mol Cancer.

[37] Wang J, Luo Y, Bi P (2020). Mechanisms of Epstein-Barr virus nuclear antigen 1 favor Tregs accumulation in nasopharyngeal carcinoma. Cancer Med.

[38] Pai S, O’Sullivan B, Abdul-Jabbar I (2007). Nasopharyngeal carcinoma-associated Epstein-Barr virus-encoded oncogene latent membrane protein 1 potentiates regulatory T-cell function. Immunol Cell Biol.

[39] Lo AK-F, Dawson CW, Lung HL, Wong K-L, Young LS. The Role of EBV-Encoded LMP1 in the NPC Tumor Microenvironment: From Function to Therapy. Front Oncol. 2021;11:262. 10.3389/fonc.2021.640207 PMC7947715 33718235

[40] Huo S, Luo Y, Deng R, et al. EBV-EBNA1 constructs an immunosuppressive microenvironment for nasopharyngeal carcinoma by promoting the chemoattraction of Treg cells. J Immunother Cancer. 2020;8(2). 10.1136/jitc-2020-001588 PMC7597532 33122398

[41] Ma J, Xuan S-H, Li Y, Zhang Z-P, Li X-H (2017). Role of the TGFβ/PDCD4/AP-1 Signaling Pathway in Nasopharyngeal Carcinoma and Its Relationship to Prognosis. Cell Physiol Biochem.

[42] Ogiya R, Niikura N, Kumaki N (2016). Comparison of tumor-infiltrating lymphocytes between primary and metastatic tumors in breast cancer patients. Cancer Sci.

